# Trust, trust repair, and public health: a scoping review

**DOI:** 10.3389/fpubh.2025.1560089

**Published:** 2025-06-11

**Authors:** Peter Kalulu, Aubrey Fisher, Grace Whitter, Ipek Sener, Michelle Doering, David B. Carter, Matthew Gabel, Jimin Ding, Michael Esposito, Caitlin L. McMurtry, Pradeep Sopory, Mark D. Huffman

**Affiliations:** ^1^Brown School of Social Work, Washington University in St. Louis, St. Louis, MO, United States; ^2^Department of Political Science, School of Arts and Sciences, Washington University in St. Louis, St. Louis, MO, United States; ^3^Bernard Becker Library, School of Medicine, Washington University in St. Louis, St. Louis, MO, United States; ^4^Department of Statistics and Data Science, School of Arts and Sciences, Washington University in St. Louis, St. Louis, MO, United States; ^5^Department of Sociology, University of Minnesota, Minneapolis, MN, United States; ^6^Department of Communication, Wayne State University, Detroit, MI, United States; ^7^Department of Medicine, School of Medicine, Washington University in St. Louis, St. Louis, MO, United States

**Keywords:** trust, public health, scoping review, trust repair, historical determinants

## Abstract

**Objective:**

This study investigates the scope of evidence on trust, trust repair, and public health.

**Methods:**

We identified quantitative studies that evaluated the relationship between trust or trust repair and public health from January 1990 to May 2023. Results were stratified evaluating trust as an exposure or outcome and reporting on trust repair. Data are reported on spatiotemporal trends in publications, level of trust (institutional trust, generalized trust, and interpersonal trust), types of trust measures used, objects and determinants of trust, and associations between trust and public health behaviors.

**Results:**

Among 194 included studies, most (86%, 166/194) were published after the COVID-19 pandemic and in high-income countries. Among 40 reports that evaluated trust as an outcome, most (52%) evaluated trust in government. Socioeconomic factors (*n* = 18), perceived government performance (*n* = 14), and media/information (*n* = 8) were the most common determinants overall and for institutional trust. Three reports focused on trust repair (*n* = 2) or maintenance (*n* = 1).

**Conclusion:**

This review provides a roadmap for future research on evaluating and improving trust and public health.

## Introduction

A person’s “willingness to be vulnerable to another for a given set of tasks” (1) is a fundamental, if often understated, component of a well-functioning health system. Public health actors are key participants in population welfare, constructing mandates and guidelines for civilians that, if widely adopted, can reduce threats of premature death and disability and help to reduce the burden of chronic and progressive diseases. However, no matter how well-designed a public health intervention may be, effective implementation of these sorts of “top-down” health initiatives is conditional on the public’s belief that the institutions promoting them are trustworthy and operate with one’s best interests in mind. Importantly, the tight connection between the success of generative health efforts and recipient trust spills across all nodes where health is produced: trusting relationships between patients, health systems, and healthcare providers are essential to promote adherence to healthcare professionals’ recommendations, and all heavily depend on trust (2). Similarly, trust in government, even when broadly construed, is a major factor underlying the success of health operations, since in many countries, governmental institutions are deeply involved in the delivery of care. They are also instrumental in the development and distribution of public health interventions, such as vaccines. Civilian trust is hence a necessary catalyst toward producing desirable population health outcomes writ large; intergovernmental, multilateral, and governmental organizations—such as the World Health Organization, Organization for Economic Cooperation and Development, and Centers for Disease Control and Prevention—have begun to center trust as a major priority for improving public health and health systems, yet have largely emphasized health system competency for trust promotion (3–5).

Despite the growing recognition of trust’s role in achieving favorable population health outcomes (6–8), empirical research in this area lags behind. Part of this is due to the lack of consensus about how to define and conceptualize trust as a factor in public health outcomes (8, 9). For example, empirical studies of the United States vary significantly in their choice of the object of trust (e.g., public health policy, public health policy maker, health care provider, or government official) and that object’s level of aggregation (e.g., system or individual) (2, 8). These follow more general social science conceptions of trust related to inter-personal and institutional trust. In particular, one who broadly trusts individuals in one’s society, including those they do not know personally, demonstrates “generalized” social trust. High levels of generalized trust can be beneficial for resolving social problems, maintaining solidarity, and promoting cooperation (8–10). The object of institutional trust is not a person or identifiable individuals but an abstract organization or system (10, 11), such as a national government or health care (10, 11). This form of trust is potentially consequential for public compliance with public health policies. In addition, the literature examines trust in specific contexts—e.g., the COVID-19 pandemic or during public health emergencies—that may not be comparable with one another or with more normal contexts (1, 12, 13).

Furthermore, empirical studies, particularly those using survey data, have pursued a wide range of measurement strategies (2, 9). These differences in conceptual and empirical approaches make conclusions about the level of trust and its effects on public health outcomes difficult to compare. These differences also hamper the cumulation of knowledge about how to improve or repair trust. Studies have investigated a variety of sources of change in trust. Any differences in findings about the impact of sources of trust or mistrust (e.g., historical events or the social/political context) may reflect the variation in methodological approaches rather than distinctions in their objective effects on trust. To address these gaps and examine how trust deficits might be remedied to improve population health, we conducted a scoping review with the following objectives: (1) to describe research on trust, trust repair, and public health across the world; (2) to summarize the scope and types of available evidence; (3) to clarify key concepts, including those related to determinants and objects of trust; and (4) to propose research priorities concerning trust, trust repair, and public health.

## Methods

The scoping review methods outlined by Arksey and O’Malley guided our approach to mapping relevant literature in the field and identifying gaps in existing research (14). In comparison to systematic reviews, scoping reviews enhance the understanding of complex topics by mapping them to illuminate the breadth of available evidence on a subject (15). A protocol for this scoping review was developed prior to executing the search and is reported (15). The Preferred Reporting Items for Systematic Reviews and Meta-Analyses Extension for Scoping Reviews (PRISMA-ScR) checklist guided the reporting (16, 17).

### Study selection criteria

This scoping review included empirical reports encompassing original quantitative research studies. Because the goal of the review was to map the extent, range, and nature of the quantitative evidence available in the existing literature, qualitative studies were excluded to prioritize quantitative evidence and enhance generalizability in guiding policy interventions.

To be eligible for inclusion, studies needed to evaluate the relationship between trust or trust repair and public health. Public health broadly includes concepts such as health status, prevention, promotion, surveillance, and outcomes related to health behaviors, medication uptake, screenings, diagnostics, and disease control. Using established trust-related frameworks (18, 19), we investigated trust and trust repair concepts, such as competency, character, caring, honesty, transparency, consistency, fiduciary responsibility, confidentiality, confidence, loyalty, and other related concepts retrieved from the search.

### Data sources and searches

To search the published literature, we developed trust and public health search strategies for MEDLINE, Embase, Scopus, and Web of Science, limiting them to English. The search dates were from January 1990 to May 2023. The search used a combination of standardized terms and keywords, including, but not limited to, (trust OR public confidence OR distrust) AND (public health OR mass drug administration OR community health services OR infectious disease transmission OR population surveillance OR government disease prevention programs). The search strategies are reported in Supplementary Table 1. We searched the reference lists of the included studies for additional papers.

### Study selection

Three reviewers independently screened the titles and abstracts of 1,963 unique empirical studies for inclusion using Covidence. Each article was assigned to one or two of the three reviewers to review the full texts, examine the selection criteria, and document the reasons for exclusion independently. Disagreements at both stages were resolved through consensus or with the involvement of another author.

### Data collection and synthesis

We designed a data extraction form to extract relevant information from the included reports. Extracted data included the title, publication year, journal authors, contact authors’ details, the country where the data was collected, study purpose, design, modality, funding sources, population description, sample size, sampling frame, inclusion/exclusion criteria, dependent/outcome variables, independent/predictor variables, mediators and moderators, trust measures, object(s) of trust, public health outcomes and behaviors, and overall findings. Three authors independently extracted data from 10% of the included studies in duplicate to assess agreement. Another author participated in discussions comparing the duplicate extraction data, and consensus was achieved through group discussion among the co-authors during regular team meetings.

Using the extracted information, the study team organized the included studies and corresponding data into identified themes for analysis with input from the other authors. We created figures to map results over time and space, highlighting the relationship of studies to contemporary pandemics and countries of study. To summarize and understand the connection between trust and public health, we divided reports into two categories: (1) reports that included trust as an outcome and (2) reports that included trust as an exposure. We cross-tabulated study characteristics, including study design, the modality of data collection, age group, sampling frame, trust measure, and evaluation of trust repair or maintenance with types of trust, including institutional trust, generalized trust, or multiple levels of trust. We also identified and cross-tabulated types of trust and public health behaviors and outcomes and grouped studies based on common themes.

### Ethics

This research is not considered human subjects research, so no ethical review was sought.

## Results

### Search results and characteristics of included reports

The study flowchart is shown in Supplementary Figure 1. The search included four databases, yielding 4,114 peer-reviewed reports published between January 1990 and May 2023. We removed 2,151 duplicate reports and screened the titles and abstracts of 1,963 unique reports. After eligibility of these reports, we excluded 1,288 that did not meet the inclusion criteria and identified and retrieved 675 full-text reports. After full-text reviewing, 481 studies were excluded, and the reasons for exclusion are reported in Supplementary Table 2. Finally, 194 reports that quantitatively evaluated the association between trust and public health behaviors and outcomes were included in the scoping review. Of these, 40 reports examined trust as an outcome, while 154 reports considered trust as a predictor (*n* = 194 total). The characteristics of the included studies are shown in Supplementary Table 3.

### Temporal trends in trust and public health research

**Figure 1:**
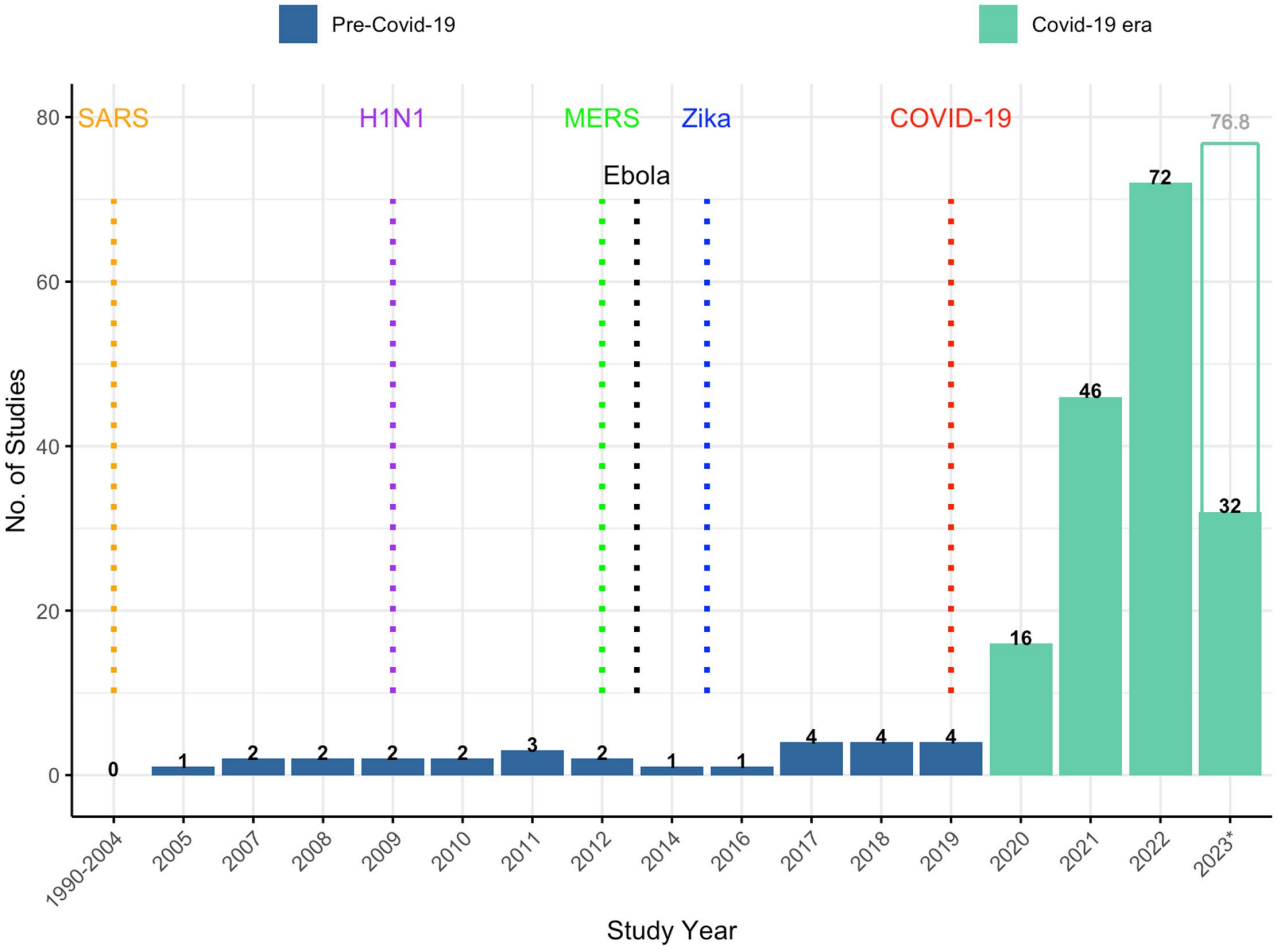
Temporal trends in trust and public health publications, 1990–2023.

Figure 1 shows temporal trends in trust and public health research over the study period. Most (86%) publications were published after the declaration of the COVID-19 pandemic. Prior to 2020, there were far fewer published reports on trust associated with earlier pandemics (20). For instance, the number of reports around the time of the Zika virus outbreak (*n* = 9), H1N1 influenza pandemic (*n* = 7), Severe Acute Respiratory Syndrome (SARS) outbreak (*n* = 5), Middle East Respiratory Syndrome (MERS) outbreak (*n* = 2), and Ebola virus outbreak (*n* = 1) is dwarfed by the approximately 170 reports published during the COVID-19 era.

### Geographical distribution of trust and public health research

Supplementary Figure 2 shows a heat map of the relative geographic distribution of the included study reports. Of the 194 reports included, some (*n* = 34) were conducted in more than one country. Therefore, in the heat map, and for each country, we report the number of times a country is mentioned in each report, resulting in a total that exceeds 194. Studies were most frequently conducted in North America, Asia, Europe, and Oceania than in other regions, while South America and Africa had the fewest number of reports. At the country level, the United States (*n* = 69), China (*n* = 37), the United Kingdom (*n* = 27), and Germany (*n* = 21) had the highest number of report, with fewer reports from Italy (*n* = 19), Sweden (*n* = 16), Australia, France, Japan, and South Korea (*n* = 15 reports each); Turkey (*n* = 13); Nigeria (*n* = 4); Kenya (*n* = 3); Uganda (*n* = 3); Ecuador (*n* = 3); Zimbabwe (*n* = 2); Bhutan (*n* = 2); Bahrain (*n* = 2); Cyprus (*n* = 2); Albania (*n* = 2); Cuba (*n* = 2); Haiti (*n* = 2); Fiji (*n* = 2); Samoa (*n* = 2); Peru (*n* = 2); Venezuela (*n* = 2); and other countries, with an average of 3.9 studies reported among each included country.

### Trust as an outcome

#### Characteristics of studies with trust as an outcome

Table 1 presents the characteristics of 40 reports that evaluated different types of trust as outcomes. Most reports focused on institutional trust (*n* = 34), with fewer reports on generalized trust (*n* = 2) or multiple types of trust (*n* = 4). More than three-fourths (78%) of reports used a cross-sectional study design, and more than half (53%) used an online platform for data collection. Most (53%) reports employed representative sampling techniques. Nearly two-thirds (65%) of the trust measures used had not been validated. Trust measures used in the reports are listed in Supplementary Table 4, stratified by measures to assess institutional trust, generalized trust, and interpersonal trust. Some researchers asked broad questions such as, “How much do you trust your country’s government to take care of its citizens?” to assess levels of public trust in the government as a whole (21). Others specified the level of government or officials as the object of trust, such as the national government, local government, health systems, public health authorities, and scientists. Only three reports focused on trust repair (*n* = 2) or trust maintenance (*n* = 1).

**Table 1:** Characteristics of studies with trust as outcome (*n* = 40).

Characteristics of studies	Types of trust as outcome			Total [*n* = 40; (%)]
	Institutional trust (*n* = 34)	Generalized trust (*n* = 2)	Multiple types of trust (*n* = 4)	
Study design				
Cross-sectional	25	2	4	31 (78)
Longitudinal	7	0	0	7 (18)
Multiple	2	0	0	2 (5)
Study modality				
Online	19	0	2	21 (53)
In-person	4	0	0	4 (10)
Mail delivery	2	0	1	3 (8)
Telephone-based	2	0	0	2 (5)
Multiple	1	0	1	2 (5)
Not reported	6	2	0	8 (20)
Age group				
Adults and children	22	2	4	28 (70)
20 years and above	7	0	0	7 (13)
Other groups	5	0	0	5 (18)
Sampling frame				
Representative	17	2	2	21 (53)
Nonrepresentative	17	0	2	19 (48)
Trust measure validated				
No	20	2	4	26 (65)
Yes	14	0	0	14 (35)
Trust repair or maintenance				
Trust repair	2	0	0	2 (5)
Trust maintenance	1	0	0	1 (3)
No intervention	31	2	4	37(92)

This table includes only studies that evaluated trust as an outcome (*N* = 40). In the study design section, “multiple” refers to cross-sectional and longitudinal designs used in a single study. In the study modality section, “multiple” refers to multiple data collection methods. “Other groups” under the age group include studies analyzed at country levels, age groups with one theme, and those that did not mention the age group of respondents. We did not independently identify interpersonal trust as an outcome.

#### Objects of trust

Supplementary Figure 3 shows the proportion of objects of trust among the 40 reports, whether as a single object or as two or more. Most reports (52%) evaluated trust in government in general. Approximately one-quarter (24%) of reports separately assessed two or more objects of trust in a single report, most commonly the government plus another object of trust. Less than 10% of reports focused on other objects of trust (e.g., public health authorities [7%], science/scientists [7%], media/information sources [5%], neighborhood/family/friends/community [3%], and health systems [2%]).

#### Predictors of trust, by type of trust

Table 2 shows the frequency of predictors of trust as outcomes by type of trust for 40 reports. Studies sometimes involved more than one predictor of trust or type of trust. Predictors such as socio-economic factors (*n* = 18), perceived government performance/quality (*n* = 14), and media and information (*n* = 8) were the most common determinants overall and for institutional trust. Self-reported health status and happiness (*n* = 1) and self-reported discrimination (*n* = 1) were the only predictors consistently associated with generalized trust. Socioeconomic factors (*n* = 3) and psychosocial factors (*n* = 3) were associated with multiple types of trust.

**Table 2:** Predictors of trust, by type of trust.

Predictor	Types of trust (*N* = 40)		
	Institutional trust	Generalized trust	Multiple types of trust
Socio-economic factors (*n* = 18)	15	0	3
Perceived government performance/quality (*n* = 14)	14	0	0
Media and Information (*n* = 8)	8	0	0
Political attitudes and orientations (*n* = 7)	6	0	1
Self-reported health status and happiness (*n* = 7)	5	1	1
Trust in other institutions (*n* = 5)	5	0	0
Health-related knowledge and attitudes (*n* = 4)	4	0	0
Government response measures (e.g., contact tracing) (*n* = 2)	2	0	0
Pandemic-related threats (*n* = 2)	2	0	0
Air pollution (e.g., PM_2.5_ concentration, cigarette smoking) (*n* = 2)	2	0	0
Health system inputs (e.g., number of hospital beds) (*n* = 2)	2	0	0
Economic struggle and financial loss during a pandemic (*n* = 2)	2	0	0
Psychosocial factors (*n* = 4)	1	0	3
Self-reported discrimination (*n* = 2)	1	1	0
Others (*n* = 9)	9	0	0

Each cell reports the number of studies that are applicable. The “Others” category includes studies for which we found no other commonly themed studies on the determinants. The numbers represent the frequency of a determinant’s mention in a report and could be more than the number of reports included in this review.

#### Cross-tabulation between trust and public health behaviors

##### Trust as a predictor

We evaluated 154 reports on the association between trust and public health behaviors and outcomes. Table 3 reports how often the relationship between different types of trust as a predictor and specific public health behaviors or outcomes was identified. The reports primarily evaluated trust in institutions, particularly in the government (*n* = 110), with questions such as “In general, how much do you trust the government of your country to take the right measures to deal with the coronavirus pandemic? (13)” and other questions included in Supplementary Table 4, health systems (*n* = 31), and media or information sources (*n* = 27), on public health behaviors or outcomes such as vaccination uptake and intention (*n* = 100). For example, reports evaluated vaccination uptake and intention for vaccination against COVID-19 (*n* = 96), influenza (*n* = 2), Ebola (*n* = 1), pertussis, measles, and influenza (*n* = 1), as well as preventive measures (*n* = 85), such as handwashing, wearing a mask, maintaining social distance, and self-reported mental and physical health and life satisfaction (*n* = 17). Less commonly, reports involved generalized trust and interpersonal trust. Generalized trust was primarily studied with respect to vaccination uptake and intention (*n* = 8), preventive measures (*n* = 8), and self-reported health and life satisfaction (*n* = 6). Finally, interpersonal trust was studied as a predictor of preventive measures (*n* = 2), such as wearing a mask and social distancing, in contrast to the lack of reports assessing interpersonal trust as an outcome.

**Table 3:** Types of trust as a predictor of public health behaviors and outcomes (*n* = 154).

Type of Trust	Public Health Behaviors and Outcomes										
	Vaccination uptake and intention	Preventive measures	Self-reported health and life satisfaction	Perceptions of public health crises and policies	Objective health outcomes	Healthcare disruptions and ART adherence	Risky behaviors and behavioral adjustments during crises	Public support for evidence-informed policymaking	Climate change policy support & environmental satisfaction	State fragility during crisis and crisis management	Others
Institutional trust											
Trust in government (*n* = 110)	39	41	5	4	3	2	2	1	2	2	9
Trust in health systems (*n* = 31)	15	9	3	1	0	1	0	0	0	0	2
Trust in media/information sources (*n* = 27)	14	5	1	1	2	2	0	0	0	0	2
Trust in public health authorities (*n* = 14)	8	1	1	1	0	1	0	0	0	0	2
Trust in science/scientists (*n* = 17)	5	10	0	0	0	0	0	1	0	0	1
Trust in other institutions (*n* = 25)	10	9	0	1	1	0	1	0	0	0	3
Generalized trust (*n* = 29)	8	8	6	1	2	0	1	0	0	0	3
Interpersonal trust (*n* = 6)	1	2	1	0	1	0	1	0	0	0	0
Total (*N*)	100	85	17	9	9	6	5	2	2	2	22

The “Others” category includes studies for which we found no other commonly themed studies on public health outcomes or behaviors (e.g., information seeking, late HIV diagnosis rates, mortality, and self-efficacy). The numbers in this table are not just counts; they represent the frequency of a determinant’s mention in a report and can be more than the number of reports included in this review. ART: Antiretroviral Therapy.

## Discussion

This scoping review identified 194 quantitative empirical reports examining trust, trust repair, and public health. The findings revealed increased publications on trust and public health over time, with a significant rise following the COVID-19 pandemic and fewer reports related to H1N1, SARS, and Zika virus outbreaks (22–31). The geographic distribution of these reports was uneven, with most focused on North America, Europe, and Asia. Longitudinal designs to understand changes in public trust over time were rare despite their importance (29, 31–38). Competence, as assessed through government performance, was associated with institutional trust, aligning with dominant trust models (39–42).

The current review extends previous work by the Centers for Disease Control and Prevention, which limited its focus to studies conducted in the United States (8), through its international perspective. Similar to the unpublished CDC scoping review, which is no longer accessible from the CDC’s website, cross-sectional surveys were predominant, particularly during the COVID-19 pandemic, when trust was identified as a critical issue. For instance, Bollyky et al. found that institutional and interpersonal trust were more influential on SARS-CoV-2 infection rates and COVID-19 vaccination uptake than pre-pandemic preparedness indices or measures of healthcare capacity (43). This and other work suggest that significant investments in trust maintenance and repair could have substantial public health benefits, although the effectiveness of specific strategies remains uncertain. An effective trust-building strategy could build on evidence that individuals’ trust in government increases in localities that are targeted by large-scale and fairly popular state investments in infrastructure, such as the electricity grid or fortifications associated with border security (44, 45). Research is needed to identify whether similar public health-related investments might also enhance institutional trust.

This review shows that most research to date has focused on communicable diseases, overlooking the growing burden of non-communicable diseases like cardiovascular diseases, cancer, obesity, and diabetes, among other conditions (2). These public health priorities could present opportunities for leveraging trust, given reports of agreement with these priorities, even among individuals with low trust in the Centers for Disease Control and Prevention or state or local public health institutions (46). Reports often concentrated on medical countermeasures (e.g., vaccines, masks) and ignored other public health functions such as assessment, assurance, and policy development (47). The previous review included additional studies evaluating interventions that may influence trust; yet, these were generally case studies, discussions, or reviews without empirical trust measurements (8).

Several reports underscored the critical link between public trust and health behaviors (22, 33, 48–51), highlighting the complexity of measuring and enhancing trust in diverse systems (52). Enhancing trust in public health systems may require fostering cooperative relationships and shared goals, whereas building trust in government may require using information technology and social media to engage citizens (35, 41, 52). Historical determinants of trust were uncommonly reported and primarily on the individual level (e.g., discrimination) (24, 45–52). Conversely, macro-level determinants, such as border instability or carceral violence, were not typically reported and likely play important roles in understanding why trust varies or is low (53, 54).

While there is a growing body of evidence on trust and public health behaviors and outcomes based on the temporal trends identified from this review, significant gaps remain. Research on trust and public health has also been limited in regions like Africa and South America. Despite the recent emphasis on enhancing trust to improve public health outcomes (6, 7), most studies do not use a longitudinal design to examine changes in trust over time, making it difficult to track how any modifications in policy or interventions might affect trust, especially on a within-individual level. Moreover, many studies used trust measures that lacked validation, undermining the reliability of reported findings. Trust assessments often focused on institutions, particularly national governments (51, 55, 56), and tended to neglect lower levels of government as well as community and interpersonal trust (39, 48). Reports on trust maintenance, building, and repair were minimal (35, 41, 52), hindering the field’s understanding of trust at different socioecological levels and its influence on adherence to public health policies.

### Recommendations and research priorities

Future efforts must adopt a proactive approach to trust maintenance, building, and repair to strengthen public health outcomes rather than waiting for crises like COVID-19 to highlight public health system vulnerabilities. History shows that low institutional trust can exacerbate health crises, leading to preventable illness and mortality. Therefore, investments in sustainable trust-building initiatives are needed to avoid future pitfalls. Future research should engage a broader range of public health functions beyond countermeasures and explore the relationship between trust and health outcomes beyond pandemics. Collaborative efforts are necessary to enhance global health research, especially in regions with low institutional trust.

Research should use longitudinal designs to track changes in trust over time and employ validated trust measures to ensure accuracy. Future research should incorporate historical determinants associated with institutional and generalized trust, including accounting for variability in interpersonal trust due to factors such as differences in individuals’ social networks. To understand how trust varies over time and space and to identify effective strategies for maintaining, building, and repairing trust for better health outcomes, there is a need to employ quantitative, qualitative, and community-based participatory approaches, including measures of trust behaviors. By fostering resilience in public health systems, they can be better prepared for unforeseen challenges and achieve improved public health behaviors and outcomes. Visible and meaningful state investment in the treatment of common non-communicable diseases, such as heart disease or diabetes, might be a promising route for trust-building or trust repair (44, 45).

### Strengths and limitations

This scoping review has several strengths, including robust search, screening, and data extraction methods that extend previous research in this area. However, it also has limitations. Only quantitative, original research studies were included, differing from other reviews on the topic. The search was restricted to reports from January 1990 to May 2023 and publications in English, which may have excluded relevant non-English studies. Although a manual search of reference lists was conducted, some studies may have been missed.

## Conclusion

This scoping review provides an overview of the evidence linking trust, trust repair, and public health. It highlights critical gaps and research priorities, such as the need for diverse study designs, validated trust measures, and incorporation of historical trust determinants. These findings can guide efforts to conceptualize trust in public health and develop strategies for trust maintenance, building, and repair within public health systems.

## Data Availability

The original contributions presented in the study are included in the article/Supplementary material, further inquiries can be directed to the corresponding author.

## References

[1] Sopory P Novak JM Day AM Eckert S Wilkins L Padgett DR. Trust and public health emergency events: a mixed-methods systematic review. Disaster Med Public Health Prep. (2022) 16:1653–73. doi: 10.1017/dmp.2021.105, PMID: 34112272

[2] Taylor LA Nong P Platt J. Fifty years of trust research in health care: a synthetic review. Milbank Q. (2023) 101:126–78. doi: 10.1111/1468-0009.12598, PMID: 36689251 PMC10037697

[3] House Committee on Energy and Commerce (2024). Health Subcommittee Hearing: “Are CDC’s Priorities Restoring Public Trust and Improving the Health of the American People?” Available online at: https://energycommerce.house.gov/events/energycommerce.house.gov (Accessed November 9, 2024).

[4] OECD. (2024). Trust in government. Available online at: https://www.oecd.org/en/topics/trust-in-government.html (Accessed November 9, 2024).

[5] WHO Initiative on Trust and Pandemic Preparedness. (2023). Available online at: https://www.who.int/initiatives/who-initiative-on-trust-and-pandemic-preparedness (Accessed November 9, 2024).

[6] Buckley C. (2023). Edelman trust barometer special report trust and health. Available online at: https://www.edelman.com/trust/2023/trust-barometer/special-report-health (Accessed May 6, 2025).

[7] COVID-19 National Preparedness Collaborators. Pandemic preparedness and COVID-19: an exploratory analysis of infection and fatality rates, and contextual factors associated with preparedness in 177 countries, from Jan 1, 2020, to sept 30, 2021. Lancet. (2022) 399:1489–512. doi: 10.1016/S0140-6736(22)00172-6, PMID: 35120592 PMC8806194

[8] Ichida A. Mezzo J Piepenbrink R. Bhalakia A. (2022). Trust in Public Health Science & Trust in Public Health Practice Scoping Review: Final report. Unpublished report submitted to the Centers for Disease Control and Prevention.

[9] Richmond J Anderson A Cunningham-Erves J Ozawa S Wilkins CH. Conceptualizing and measuring trust, mistrust, and distrust: implications for advancing health equity and building trustworthiness. Annu Rev Public Health. (2024) 45:465. doi: 10.1146/annurev-publhealth-061022-044737, PMID: 38100649 PMC11156570

[10] Giddens A. (1990). The consequences of modernity. Stanford University Press. Available online at: https://www.sup.org/books/sociology/consequences-modernity (Accessed April 27, 2025).

[11] Luhmann N. The paradox of system differentiation and the evolution of society. Differentiation theory and social change: Comparative and historical perspectives. (1990). 409–40. Available online at: https://luhmann.ir/wp-content/uploads/2021/07/The-Paradox-of-System-Differentiation-and-the-Evolution-of-Society.pdf (Accessed May 6, 2025).

[12] Gambetta D Morisi D. COVID-19 infection induces higher trust in strangers. Proc Natl Acad Sci. (2022) 119:e2116818119. doi: 10.1073/pnas.2116818119, PMID: 35917349 PMC9371727

[13] Han Q Zheng B Cristea M Agostini M Bélanger JJ Gützkow B. Trust in government regarding COVID-19 and its associations with preventive health behaviour and prosocial behaviour during the pandemic: a cross-sectional and longitudinal study. Psychol Med. (2021) 53:149–59. doi: 10.1017/S0033291721001306, PMID: 33769242 PMC8144822

[14] Arksey H O’Malley L. Scoping studies: towards a methodological framework. Int J Soc Res Methodol. (2005) 8:19–32. doi: 10.1080/1364557032000119616

[15] Kalulu P Fisher A Whitter G Doering M Carter D Gabel M. (2023). Trust, Trust Repair, and Public Health: A Scoping Review Protocol. Available online at: https://digitalcommons.wustl.edu/cgi/viewcontent.cgi?article=1005&context=data (Accessed May 6, 2025). 10.3389/fpubh.2025.1560089 PMC12199165 40575096

[16] Tricco AC Lillie E Zarin W O'Brien KK Colquhoun H Levac D. PRISMA extension for scoping reviews (PRISMA-ScR): checklist and explanation. Ann Intern Med. (2018) 169:467–73. doi: 10.7326/M18-0850, PMID: 30178033

[17] Page MJ McKenzie JE Bossuyt PM Boutron I Hoffmann TC Mulrow CD. The PRISMA 2020 statement: an updated guideline for reporting systematic reviews. J Clin Epidemiol. (2021) 134:178–89. doi: 10.1016/j.jclinepi.2021.03.001, PMID: 33789819

[18] Dirks KT de Jong B. Trust within the workplace: a review of two waves of research and a glimpse of the third. Annu Rev Organ Psych Organ Behav. (2022) 9:247–76. doi: 10.1146/annurev-orgpsych-012420-083025

[19] Lewicki RJ Brinsfield C. Trust repair. Annu Rev Organ Psych Organ Behav. (2017) 4:287. doi: 10.1146/annurev-orgpsych-032516-113147, PMID: 39807870

[20] Bhadoria P Gupta G Agarwal A. Viral pandemics in the past two decades: an overview. J Family Med Prim Care. (2021) 10:2745–50. doi: 10.4103/jfmpc.jfmpc_2071_20, PMID: 34660399 PMC8483091

[21] Stanica C Crosby A Larson S. Trust in government and COVID-19 response policy: a comparative approach. J Comp Policy Anal. (2023) 25:156–71. doi: 10.1080/13876988.2022.2103672

[22] Gotanda H Miyawaki A Tabuchi T Tsugawa Y. Association between trust in government and practice of preventive measures during the COVID-19 pandemic in Japan. J Gen Intern Med. (2021) 36:3471–7. doi: 10.1007/s11606-021-06959-3, PMID: 34159544 PMC8218973

[23] Lim VW Lim RL Tan YR Soh AS Tan MX Othman NB. Government trust, perceptions of COVID-19 and behaviour change: cohort surveys, Singapore. Bull World Health Organ. (2021) 99:92–101. doi: 10.2471/BLT.20.269142, PMID: 33551503 PMC7856356

[24] Shibli H Palkin D Aharonson-Daniel L Davidovitch N Daoud N. Inequalities in trust levels and compliance with physical distancing during COVID-19 outbreaks: comparing the Arab minority and Jewish populations in Israel. Int J Public Health. (2022) 67:1604533. doi: 10.3389/ijph.2022.1604533, PMID: 35450127 PMC9017601

[25] Arvanitis M Opsasnick L O'Conor R Curtis L Vuyyuru C Yoshino J. Factors associated with COVID-19 vaccine trust and hesitancy among adults with chronic conditions. Prev Med Rep. (2021) 24:101484. doi: 10.1016/j.pmedr.2021.101484, PMID: 34306998 PMC8280610

[26] Rivera JD. Trust in government actors and COVID-19 vaccination uptake among Hispanics and Latinos in the U.S. Int J Disaster Risk Reduct. (2023) 89:103627. doi: 10.1016/j.ijdrr.2023.103627, PMID: 36909818 PMC9987608

[27] Amo-Adjei J Nurzhynska A Essuman R Lohiniva AL. Trust and willingness towards COVID-19 vaccine uptake: a mixed-method study in Ghana, 2021. Arch Public Health. (2022) 80:64. doi: 10.1186/s13690-022-00827-0, PMID: 35189963 PMC8860287

[28] Freimuth VS Musa D Hilyard K Quinn SC Kim K. Trust during the early stages of the 2009 H1N1 pandemic. J Health Commun. (2014) 19:321–39. doi: 10.1080/10810730.2013.811323, PMID: 24117390 PMC3943629

[29] Bangerter A Krings F Mouton A Gilles I Green EGT Clémence A. Longitudinal investigation of public trust in institutions relative to the 2009 H1N1 pandemic in Switzerland. PLoS One. (2012) 7:e49806. doi: 10.1371/journal.pone.0049806, PMID: 23185444 PMC3504102

[30] Van Der Weerd W Timmermans DR Beaujean DJ Oudhoff J Van Steenbergen JE. Monitoring the level of government trust, risk perception and intention of the general public to adopt protective measures during the influenza a (H1N1) pandemic in the Netherlands. BMC Public Health. (2011) 11:575. doi: 10.1186/1471-2458-11-575 21771296 PMC3152536

[31] Zhai K Yuan X Zhao G. The impact of major public health emergencies on Trust in Government: from SARS to COVID-19. Front Psychol. (2022) 13:1030125. doi: 10.3389/fpsyg.2022.1030125, PMID: 36467202 PMC9710386

[32] Yokoyama HM Ikkatai Y. Support and trust in the government and COVID-19 experts during the pandemic. Front Commun. (2022) 7:940585. doi: 10.3389/fcomm.2022.940585

[33] Bajos N Spire A Silberzan L Sireyjol A Jusot F Meyer L. When lack of Trust in the Government and in scientists reinforces social inequalities in vaccination against COVID-19. Front Public Health. (2022) 10:908152. doi: 10.3389/fpubh.2022.908152, PMID: 35937246 PMC9346080

[34] Blair RA Curtice T Dow D Grossman G. Public trust, policing, and the COVID-19 pandemic: evidence from an electoral authoritarian regime. Soc Sci Med. (2022) 305:115045. doi: 10.1016/j.socscimed.2022.115045, PMID: 35623233 PMC9122739

[35] Tanemura N Kakizaki M Kusumi T Onodera R Chiba T. Levels of trust in risk-only negative health messages issued by public agencies: a quantitative research-based mindsponge framework. Humanit Soc Sci Commun. (2022) 9:388. doi: 10.1057/s41599-022-01415-x

[36] Zhao E Wu Q Crimmins EM Ailshire JA. Media trust and infection mitigating behaviours during the COVID-19 pandemic in the USA. BMJ Glob Health. (2020) 5:e003323. doi: 10.1136/bmjgh-2020-003323, PMID: 33037063 PMC7545496

[37] Choi Y Fox AM. Mistrust in public health institutions is a stronger predictor of vaccine hesitancy and uptake than Trust in Trump. Soc Sci Med. (2022) 314:115440. doi: 10.1016/j.socscimed.2022.115440, PMID: 36332532 PMC9557136

[38] Nivette A Ribeaud D Murray A Steinhoff A Bechtiger L Hepp U. Non-compliance with COVID-19-related public health measures among young adults in Switzerland: insights from a longitudinal cohort study. Soc Sci Med. (2021) 268:113370. doi: 10.1016/j.socscimed.2020.113370, PMID: 32980677 PMC7493799

[39] Pratolo S Sofyani H Maulidini RW. The roles of accountability and transparency on public trust in the village governments: the intervening role of COVID-19 handling services quality. Cogent Bus Manag. (2022) 9:648. doi: 10.1080/23311975.2022.2110648

[40] Al-Omoush KS Garrido R Cañero J. The impact of government use of social media and social media contradictions on trust in government and citizens’ attitudes in times of crisis. J Bus Res. (2023) 159:3748. doi: 10.1016/j.jbusres.2023.113748

[41] Mansoor M. An interaction effect of perceived government response on COVID-19 and government agency’s use of ICT in building trust among citizens of Pakistan. TG. (2021) 15:693–707. doi: 10.1108/TG-01-2021-0002

[42] Hartanto D Siregar SM. Determinants of overall public trust in local government: meditation of government response to COVID-19 in Indonesian context. TG. (2021) 15:261–74. doi: 10.1108/TG-08-2020-0193

[43] Bollyky TJ Castro E Aravkin AY Bhangdia K Dalos J Hulland EN. Assessing COVID-19 pandemic policies and behaviours and their economic and educational trade-offs across US states from Jan 1, 2020, to July 31, 2022: an observational analysis. Lancet. (2023) 401:1341–60. doi: 10.1016/S0140-6736(23)00461-0, PMID: 36966780 PMC10036128

[44] Carter DB Deng RW. (2025). "How does state presence affect Trust in Hard to reach places?" Working Paper, Washington University in St. Louis.

[45] Carter DB Kenwick M Prati A Simmons B. Border fortification and Trust in Political Institutions Working Paper, Washington University in St. Louis, Rutgers University, and the University of Pennsylvania (2025).

[46] SteelFisher GK Findling MG Caporello HL Lubell KM Vidoloff Melville KG Lane L. Trust in US federal, state, and local public health agencies during COVID-19: responses and policy implications. Health Aff. (2023) 42:328–37. doi: 10.1377/hlthaff.2022.01204, PMID: 36877902 PMC11318038

[47] Center for Disease Control and Prevention. (2020). The ten essential public health services. Atlanta, GA: Center for Disease Control and Prevention. Available online at: https://phaboard.org/wp-content/uploads/EPHS-English.pdf (Accessed November 11, 2024).

[48] Briggs HE Kim I Mowbray O Orellana ER Elkins J. Trusting and dependable sibling relationships as social capital among African American youth. J Subst Use. (2018) 23:557–62. doi: 10.1080/14659891.2018.1451565

[49] Ahnquist J Wamala SP Lindstrom M. What has trust in the health-care system got to do with psychological distress? Analyses from the national Swedish survey of public health. Int J Qual Health Care. (2010) 22:250–8. doi: 10.1093/intqhc/mzq024, PMID: 20508017

[50] Mohseni M Lindstrom M. Social capital, trust in the health-care system and self-rated health: the role of access to health care in a population-based study. Soc Sci Med. (2007) 64:1373–83. doi: 10.1016/j.socscimed.2006.11.023, PMID: 17202025

[51] Ye X Lee HH Hui KH Xin M Mo PKH. Effects of negative attitudes towards vaccination in general and Trust in Government on uptake of a booster dose of COVID-19 vaccine and the moderating role of psychological reactance: an observational prospective cohort study in Hong Kong. Vaccine. (2023) 11:393. doi: 10.3390/vaccines11020393, PMID: 36851270 PMC9961443

[52] Adam MB Donelson A. Trust is the engine of change: a conceptual model for trust building in health systems. Syst Res Behav Sci. (2022) 39:116–27. doi: 10.1002/sres.2766

[53] Abramson SF Carter DB Ying L. Historical border changes, state building, and contemporary trust in Europe. Am Polit Sci Rev. (2022) 116:875–95. doi: 10.1017/S0003055421001428

[54] Anderson A Lewis DF Shafer P Anderson J LaVeist TA. Public trust is earned: historical discrimination, carceral violence, and the COVID-19 pandemic. Health Serv Res. (2023) 58 Suppl 2:218–28. doi: 10.1111/1475-6773.14187, PMID: 37279782 PMC10339167

[55] Ekwebelem O Onyeaka H Yunusa I Ekwebelem OC Miri T Onwuneme YM. Do we trust the government? Attributes of COVID-19 vaccine hesitancy and acceptance in Nigeria. AIMS Med Sci. (2022) 9:268–82. doi: 10.3934/medsci.2022010

[56] Apeti AE. Does trust in govern ment improve Covid-19’s crisis management? SN Soc Sci. (2022) 2:202. doi: 10.1007/s43545-022-00505-6, PMID: 36158179 PMC9489261

