# Supplementary material for: Trust, trust repair, and public health: a scoping review
Source: Front Public Health. 2025 Jun 11;13:1560089. doi: 10.3389/fpubh.2025.1560089 (PMC12199165; doi:10.3389/fpubh.2025.1560089)
Supplement: Supplementary file 1 [file Data_Sheet_1.pdf]

Supplemental **Figure 1**. PRISMA Flow Chart[1]

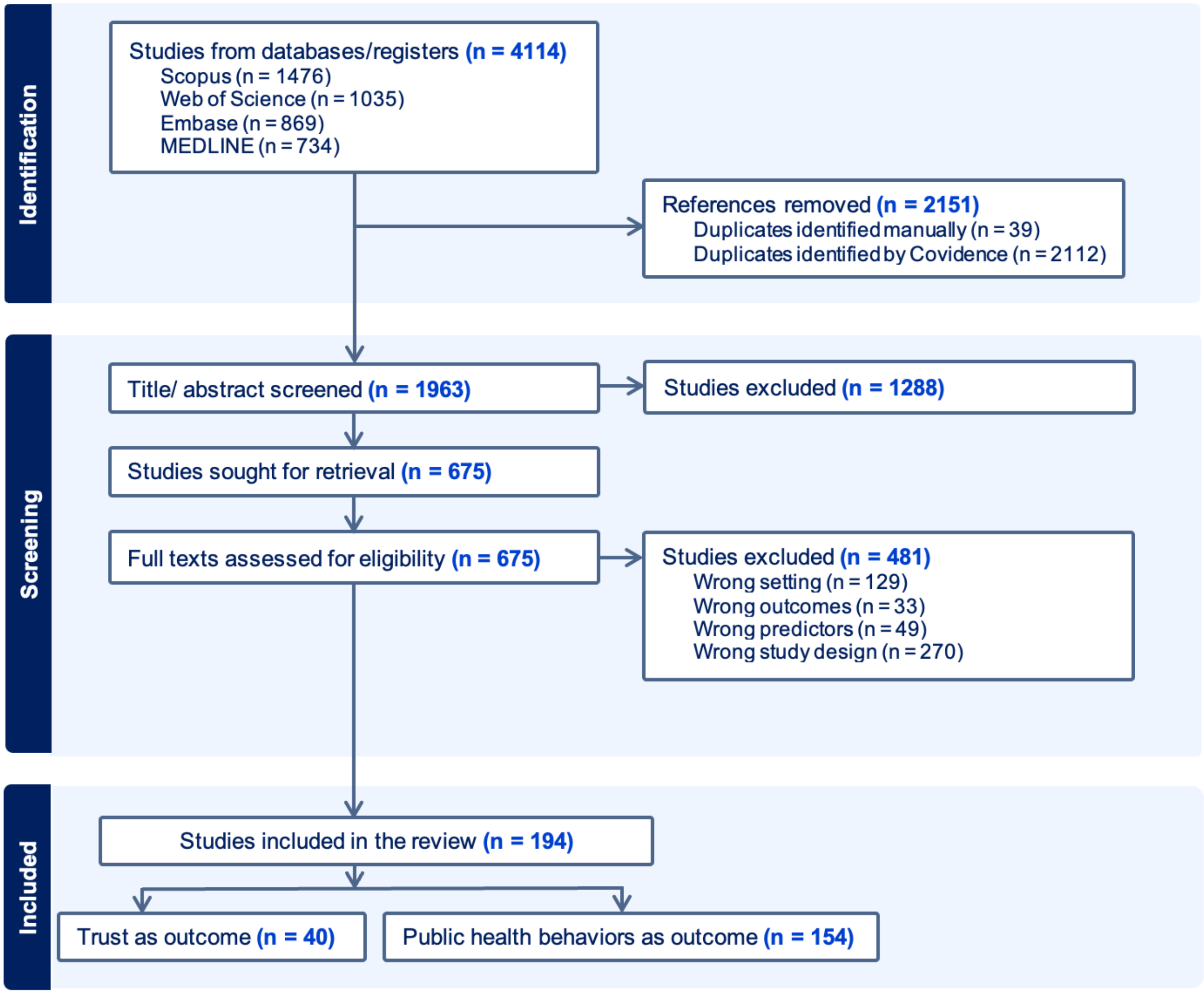

[1] We divided the reports into two main categories: those that evaluated trust as an outcome (n = 40) and those that evaluated trust on public health behaviors or outcomes (n = 154).

Supplemental **Figure 2.** Heat Map showing Included Reports by Country.[2]

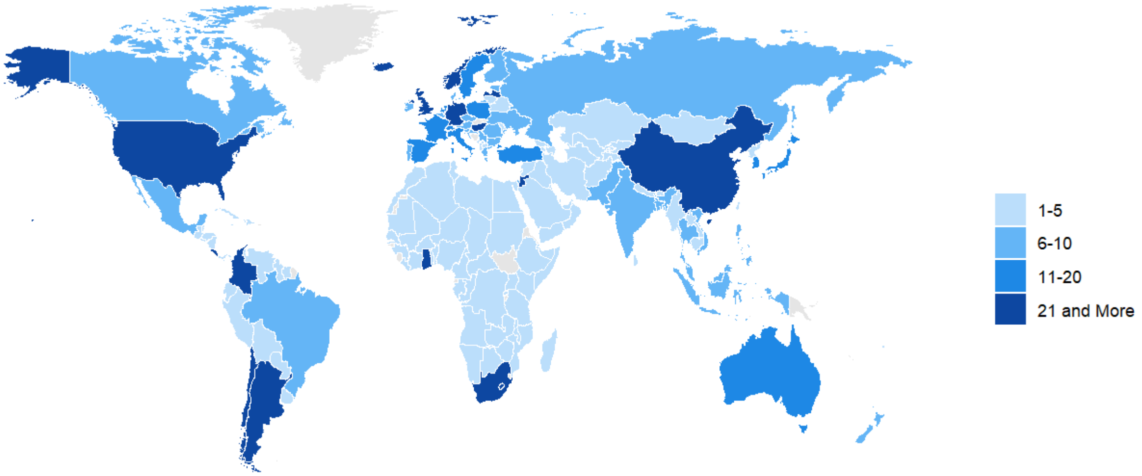

[2] The heatmap displays the count of countries mentioned and included in reports. Countries with fewer reviews (from 1) are represented by a lighter color, while countries with more reviews (up to 69) are represented by a darker color. The heatmap counts may be higher than the total number of studies in this review.

Supplemental **Figure 3**. Included objects of trust (n = 40)[3]

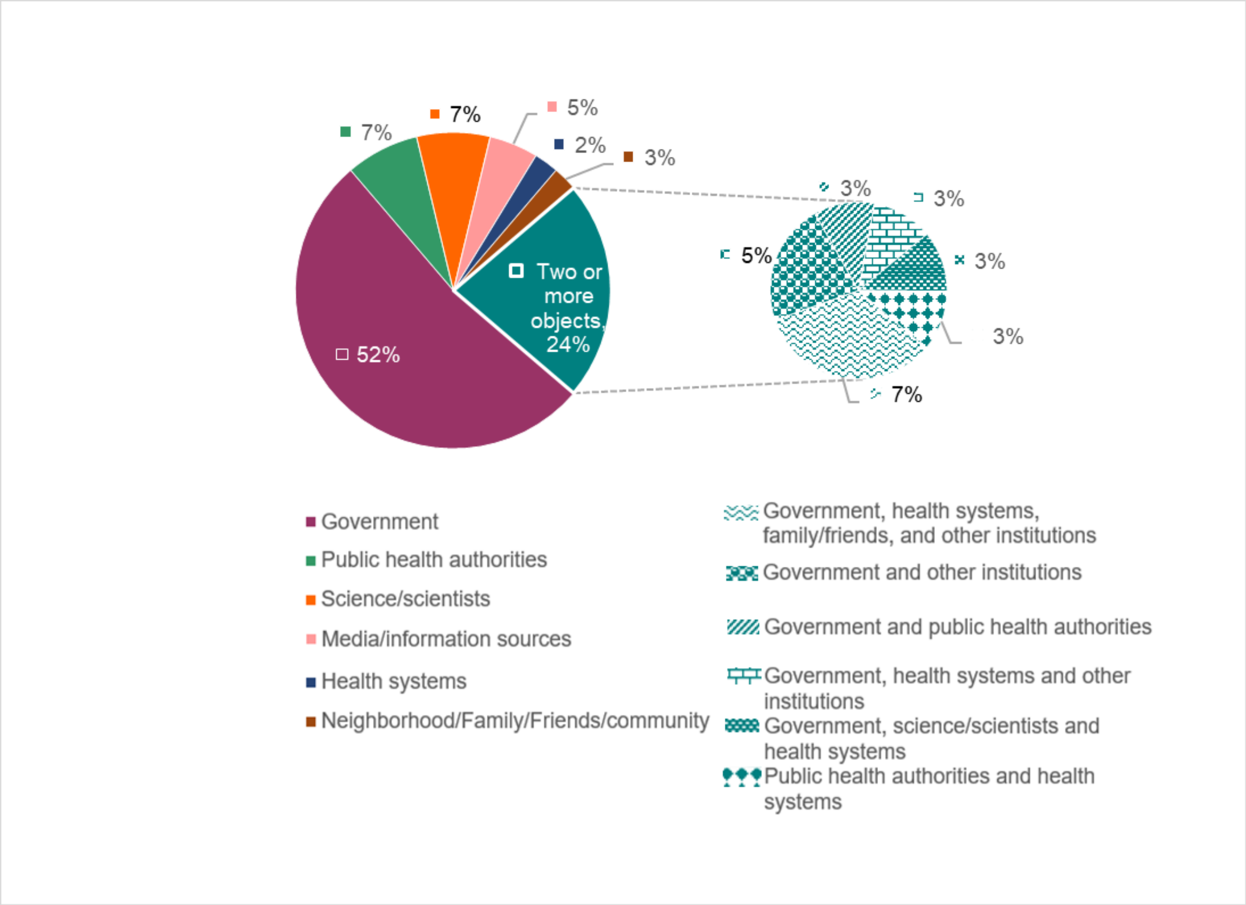

[3] The pie chart is based entirely on trust as an outcome (n = 40), and the extracted piece of the pie expands on reports that evaluated two or more types of trust in a single report.

Supplemental **Table 1**. Survey measures of trust.

Survey Question	Response Options	Number	Regions
**Trust in institutions**			
Indicate how much you agree with the following statement: I trust the (object)	Likert scale	3	Asia; Europe
How much do you trust the (object)	Likert scale	11	Global; North America; Europe; Asia
What trust do you have in (object)?	Likert scale	3	Europe
Please tell me, on a score of 0-10, how much you personally trust (institution)	Thermometer scale	5	Global; Asia; Europe
How often can you trust (institution) to do the right thing?	Likert scale	2	Global; North America
What is the level of trust you place in (object)?	Likert scale	2	Europe
Please indicate how much trust you have in the following institutions to operate in the best interest of society: (list of institutions)	Likert scale	2	Global; Europe
How much do you trust the following sources of information? (List of objects)	Likert scale	4	North America; Asia
**Generalized/ Social trust**			
Would you say that most people can be trusted, or that you can't be too careful in dealing with people?	Thermometer scale	2	Global; Europe
Generally, can you trust other people?	Likert scale	4	Asia; North America; Europe
Indicate your agreement with the following statement: People can generally be trusted	Likert scale	2	Europe
**Interpersonal trust**			
How much can you rely on/ open to your (family members, friends, peers, etc.)?	Likert scale	2	North America

Note: We include single survey items for measuring trust employed in 2 or more studies and where the survey question was explicitly presented in the report. A very small number of studies included a multi-item battery of questions to measure trust. These are not included here.

Supplemental **Table 2**. Reasons for excluded studies.

Reasons	First author (Year)
Wrong outcomes	Arthur 2023, Ballmann 2022, Bergman 2019, Cartanya-Hueso 2022, Cruwys 2021, deCarvalho 2022, Diotaiuti 2021, Eisenman 2012, Evans 2022, Fjaeran 2021, Goldfinch 2021, Gopalan 2022, Knight 2015, L'Engle 2023, Lin 2016, Liu 2023, Meyer 2013, Mitchell 2021, Mizrahi 2009, Owusu 2019, Papadopoulos 2012, Patel 2023, PaulLatupeirissa 2022, PavelaBanai 2022, Purvis 2021, Radin 2013, Rice 2022, Tan 2018, Touil 2022, Verma 2022, WebbHooper 2019, Wilson 2013, Yang 2023
Wrong predictors	Adams 2018, Akpan 2021, Arhiri 2022, Chayinska 2022, Choi 2022, deVereHunt 2022, Dhanani 2020, Durvasula 2019, Engdahl 2014, Erhardt 2021, FallahZavareh 2022, Franke 2021, Gardner 2017, Glanville 2018, Gong 2020, Han 2019, Hartanto 2021, Hassan 2020, Huang 2023, Justwan 2019, Kasstan 2022, Koetke 2021, LÄƒzÄƒroiu, 2021, Lamarche 2020, Lee 2022, Lillefjell 2018, Lowe 2022, Marlowe 2009, McClaran 2022, Mizrahi 2020, Nair 2021, Njoga 2022, O'Connor 2008, Okada 2023, Peng 2023, Reichelt 2023, Roberts 2018, Tan 2022, Travaglino 2021, Turner 2023, vanderCruijsen 2022, Wang 2022, Wynen 2022, Xie 2022, Yang 2023, Zheng 2022, Zhou 2023
Wrong settings	Abelson 2009, AnsteyWatkins 2021, Antoine-LaVigne 2023, Arcila-Calderon 2021, Asan 2021, Bocquier 2018, Bohn 2020, Cafferty 2023, Canlas 2022, Chen 2022, Cheung 2008, Christie 2022, Chu 2021, Clench-Aas 2021, Cocksedge 2019, Coveney 2008, Cuffee 2013, Cvjetkovic 2022, Dahal 2014, Defeyter 2021, Delany-Crowe 2019, Ding 2022, Dribe 2003, DubÃ© 2020, Dube 2021, Enguita-Fernandez 2021, Enria 2021, Frewer 2003, Froestad 2005, Geisterfer-Black 2022, Gopichandran 2013, Grant 2017, Gregory 2021, He 2022, Henderson 2020, Huang 2020, Huston 2009, Isaacs 2013, Jones 2018, Juarez 2022, Kikulwe 2011, KirkSell 2020, Kittelsen 2019, Kjellberg 2020, Kolner 2022, Kweekel 2017, Lago 2017, LaMarca 1996, Lanin 2019, Laor 2022, Larson 2021, Lau 2020, Lenton 2022, Lin 2022, Liu 2023,

	Lopez-Navarro 2013, Lu 2021, MacIntyre 2013, Mackay 2022, Make 2022, Makowska 2022, MartÃ-nez 2019, McKee 2009, Meftah 2019, Meredith 2007, Moucheraud 2021, Murayama 2012, Musa 2009, Myhre 2022, NegurÄƒ 2021, Noor 2022, Nour 2022, Nuriddin 2018, Nxumalo 2016, Oh 2022, Oldeweme 2021, OmidvarTehrani 2022, Oyeyemi 2023, Palmer 2009, Pang 2023, Pepper 2022, Pringle 2022, Qi 2021, Qiao 2022, Quinn 2013, Raude 2016, Rennie 2020, Roberts 2023, Ryan 2019, SadeghiBazargani 2020, Samuels 2008, Sheikh 2015, Shelton 2021, Sillence 2019, Simas 2020, Simas 2021, SnieÄ□kutÄ— 2021, Soveri 2021, Sripad 2022, Sudhipongpracha 2018, Sullivan 2022, Tabler 2022, Tang 2013, Tokuda 2011, Treloar 2016, Truong 2023, Usman 2019, Veinot 2013, Vereen 2020, Verger 2018, ViskupiÄ□ 2023, Wang 2022, Ward 2017, Ward 2021, Warren 2020, Watermeyer 2022, Weber 2023, Wei 2022, Wei 2023, Whetten 2006, Wu 2008, Wu 2022, Yuan 2021, Zhai 2022, Zhang 2022, Zohar 2022, Peek 2013.
Wrong design	Achmad 2022, Aechtner 2022, Agley 2021, Ahmad 2022, Ahmed 2021, Å½eÅ¾elj 2023, Akula 2000, Albanese 2022, Algan 2021, Allen 2018, Allington 2022, Ammon 2018, Anand 2015, Anderson 2017, Anderson 2021, Andersson 2023, Antin 2021, Arakelyan 2021, Arimoto 2012, Arriola 2021, Ashton 2019, AuBuchon 1999, Auckland 2022, Awuni 2022, Aynalem 2022, Badur 2020, Benjamin 2021, Bernadas 2021, Best 2021, Birungi 1998, Blair 2017, Blendon 2022, Bodas 2021, Boufides 2019, Bourguignon 2021, Bowman 2017, Brandt 2015, Broadbent 2020, Brown 2002, Brown 2021, Brown-Johnson 2018, Brun 2022, Bunton 2013, Burnett 2005, Cairns 2013, Casara 2022, Chairil 2020, Chan 2021, Chang 2022, Chanley 2000, Chen 2021, Childress 2008, Christie 2021, Chukwuma 2019, Claeys 2022, Cole 2021, Cummings 2014, Dal 2022, Dalton-Brown 2023, Danish 2022, Dasandi 2021, DeBlasio 2020, Devine 2023, deVries 2023, Ducci 2022, Eisenstein 2014, Elgar 2010, Englund 2022, EteroviÄ‡ 2022, Ezezika 2015, Fell 2021, Flew 2021, Ford 2009, Fu 2019, George 2022, Gilbert 2005, Gille 2017, Gin

	2022, Giordano 2019, GonzÃ¡lez-Melado 2021, Gonzalez-Melado 2021, Gopez 2021, Gopichandran 2017, Gopichandran 2020, Gottlieb 2009, Gresham 2009, Grieb 2021, Halsey 2017, Hanson 2022, Harring 2021, Hatala 2022, Hatton 2022, Helsingen 2020, Herrero-Arias 2022, Hickman 2009, Holroyd 2020, Huber 2019, Hunter 2023, Hwang 2020, Isaacs 2012, Jagosh 2015, Jamison 2019, Jhang 2020, Johns 2010, Kanovsky 2020, Karabela 2021, Katz 2022, Kazemian 2021, Keelan 2008, KibonganiVolet 2022, Kim 2016, Kim 2020, Kim 2022, Kirkman-Liff 2003, Kunitoki 2021, Lalot 2022, Lalot 2023, Lang 2021, Langwerden 2022, Lau 2020, Laursen 2018, Lazarus 2022, Ledford 2022, Lee 2019, Lee 2021, Lencucha 2021, Levitt 2022, Li 2021, Liao 2014, Limaye 2020, Lin 2022, LindstrÃ¶m 2008, Lister 2023, Liu 2023, Lovari 2020, Ma 2019, Mackay 2022, Majid 2021, Mankell 2023, Martinez-Bravo 2022, McCaughey 2017, McPherson 2001, Melovic 2020, Meyer 2008, Miyachi 2020, Mizrahi 2020, Mkhize 2023, Molster 2013, Muhammad 2021, Mumby 2017, Murtagh 2022, Musa 2022, Nagler 2023, Narayan 2021, Negri 2022, Newton 2020, Nickel 2022, Nielsen 2021, NihlenFahlquist 2018, Nong 2021, Nurutdinova 2021, Offerdal 2021, Ojikutu 2021, Oliver 2009, Opel 2020, Orobaton 2007, OudeGroeniger 2021, Oza 2023, Palanisamy 2018, Park 2020, Park 2021, Pfortner 2022, Phommasack 2013, Plohl 2022, Postill 2022, Pramiyanti 2020, Prati 2011, Pronk 2023, Ratzan 2007, Reiersen 2022, Reynolds 2008, Reynolds 2010, Reynolds 2022, Richards 2019, Rieger 2022, Rothstein 2005, Rump 2021, Rushton 2014, Sacks 2022, Saechang 2021, Salonen 1996, Sanford 2020, Sapienza 2023, Saulsberry 2013, Scherer 2016, Schiavo 2022, Schmeisser 2021, Schmelz 2020, Shah 2022, Sibley 2020, Siegrist 2001, Siegrist 2014, Siegrist 2021, Simon 2021, Smith 2005, Sondagar 2020, South 2012, Stacey 2021, Stasik 2022, Stefaniak 2022, Sutton 2018, Tabong 2022, Tan 2022, Taylor-Gooby 2006, Tetteh 2022, Thomas 1991, Thomas 2001, Thompson 2023, Thornton 2022, Trent 2022, Trivedi 2020, Vaala 2022, vanDijck 2020, Vardavas 2021, Vergara

	2021, Voils 2005, Wang 2018, Wang 2022, Whembolua 2020, Williams 2007, Winters 2022, WorldHlthOrganWorGrpPreIn 2004, Wright 2021, Wynia 2006, Wynia 2007, Xue 2021, Ye 2011, Yellinek 2021, Yuan 2022, ZeegersPaget 2016, Zhang 2023, Zulu 2022, Sripad 2021

Supplemental **Table 3**. Search strategies.

Database	Search Number	Number of Ti/Abs	Search strategy
Scopus	1	1476	(TITLE-ABS-KEY ((Trust or public-confidence) w/8 (repair* or improv* or strateg* or mitigat* or rebuil* or build* or built OR restor* or intervention* or program* or framework* or implement* or engender* or create* or bring* or invoke* or beget* or establish* or foster* or cultivat* or nurture* or nourish or obtain* OR generate* or induce* or yield* or introduce OR introducing or develop or render* or begin* or produce* or effectuate* or catalyze* or encourage* or contribute* or determine* or launch* OR enhanc*))) OR (TITLE-ABS-KEY ((Trust or public-confidence) w/3 (promote* or result* or effect* or cause*))) OR (TITLE-ABS-KEY ((mistrust OR trust OR distrust) w/2 (historical OR government OR public-health OR community-health)))
	2		(TITLE-ABS-KEY (health w/3 (public OR community OR international OR national OR government* OR promotion* OR Upstream))) OR (TITLE-ABS-KEY (disease w/6 (eliminat* OR eradicat*))) OR (TITLE-ABS-KEY (immunization* OR immunisation* OR vaccination* OR Communicable-disease-control* OR mass-screening)) OR (TITLE-ABS-KEY (Flatten w/3 epidem*)) OR (TITLE-ABS-KEY (government w/4 (pandemic OR covid-19 OR disease-control OR disease-prevention)))
	3		(TITLE (trust OR public-health))
	4		#1 AND #2 AND #3 AND (LIMIT-TO (SRCTYPE , "j") OR LIMIT-TO (SRCTYPE , "d") OR LIMIT-TO (SRCTYPE , "Undefined")) AND (LIMIT-TO (LANGUAGE , "English"))
Web of Science	1	1035	TS= ((Trust or public-confidence) near/8 (repair* or improv* or strateg* or mitigat* or rebuil* or build* or built OR restor* or intervention* or program* or framework* or implement* or engender* or create* or bring* or invoke* or beget* or establish* or foster* or cultivat* or nurture* or nourish or obtain* OR generate* or induce* or yield* or introduce OR introducing or develop or render* or begin* or produce* or

			effectuate* or catalyze* or encourage* or contribute* or determine* or launch* OR enhanc*)) OR TS= ((Trust or public-confidence) near/3 (promote* or result* or effect* or cause*)) OR TS= ((mistrust OR trust OR distrust) near/2 (historical OR government OR public-health OR community-health))
	2		TS= (health near/3 (public OR community OR international OR national OR government* OR promotion* OR Upstream)) OR TS= (disease near/6 (eliminat* OR eradicat*)) OR TS= (immunization* OR immunisation* OR vaccination* OR Communicable-disease-control* OR mass-screening) OR TS= (Flatten near/3 epidem*) OR TS= (government near/4 (pandemic OR covid-19 OR disease-control OR disease-prevention))
	3		TI= (trust OR public-health)
	4		#1 AND #2 AND #3 AND (LIMIT-TO (SRCTYPE , "j") OR LIMIT-TO (SRCTYPE , "d") OR LIMIT-TO (SRCTYPE , "Undefined")) AND (LIMIT-TO (LANGUAGE , "English"))
Ovid Medline	1	797	(((Trust or public-confidence) adj3 (repair* or improv* or strateg* or mitigat* or rebuil* or build* or built or restor* or intervention* or program* or framework* or implement* or engender* or create* or bring* or invoke* or beget* or establish* or foster* or cultivat* or nurture* or nourish or obtain* or generate* or induce* or yield* or introduce or introducing or develop or render* or begin* or produce* or effectuate* or catalyze* or encourage* or contribute* or determine* or launch* or enhanc*)) or ((Trust or public-confidence) adj3 (promote* or result* or effect* or cause*)) or ((mistrust or trust or distrust) adj2 (historical or government or public-health or community-health))).mp.
	2		Public health/ or exp public health practice/ or exp disease eradication/ or exp mass drug administration/ or exp health promotion/ or exp health priorities/ or exp community health services/ or exp immunization programs/ or exp environmental Medicine/ or Epidemiologic methods/ or Contact Tracing/ or exp population surveillance/ or exp Behavioral Risk Factor Surveillance System/ or exp Infectious Disease

			Transmission/ or Emergencies/ or exp preventive health services/ or (disease adj6 (eliminat* or eradicat*)).mp. or Communicable-disease-control*.mp. or mass-screening.mp. or (health adj3 (public or community)).mp. or (Flatten adj3 epidem*).mp. or public-trust.mp. or (government adj4 (pandemic or covid-19 or disease-control or public or preventive or preventative)).mp. or (health adj4 public).jw.
	3		trust.ti. 9664
	4		public health.ti. 65132
	5		3 or 4 74717
	6		1 and 2 and 5 820
	7		limit 6 to english language 797
Embase.com	1	869	(((Trust or public-confidence) near/8 (repair* or improv* or strateg* or mitigat* or rebuil* or build* or built OR restor* or intervention* or program* or framework* or implement* or engender* or create* or bring* or invoke* or beget* or establish* or foster* or cultivat* or nurture* or nourish or obtain* OR generate* or induce* or yield* or introduce OR introducing or develop or render* or begin* or produce* or effectuate* or catalyze* or encourage* or contribute* or determine* or launch* OR enhanc*)) or ((Trust or public-confidence) near/3 (promote* or result* or effect* or cause*))):ti,ab,kw,de OR ((mistrust OR trust OR distrust) near/2 (historical OR government OR public-health OR community-health)):ti,ab,kw,de
	2		'public health'/exp OR ‘disease elimination’/exp OR 'mass drug administration'/exp OR 'communicable disease control'/exp OR ‘public health campaign’/exp OR ‘health care planning’/exp OR ‘preventive health service’/exp OR ‘community care’/de OR ‘community program’/de OR ‘Contact examination’/exp OR ‘behavioral risk factor surveillance system’/exp OR (health near/3 (public OR community OR international OR national OR government* OR promotion* OR Upstream)):ti,ab,kw,de OR (disease near/6 (eliminat* OR eradicat*)):ti,ab,kw,de OR Communicable-disease-control*:ti,ab,kw,de OR mass-screening:ti,ab,kw,de OR (Flatten near/3 epidem*):ti,ab,kw,de OR public-trust:ti,ab,kw,de

			OR immunization*:ti,ab,kw,de OR immunisation*:ti,ab,kw,de OR vaccination*:ti,ab,kw,de OR (government near/4 (pandemic OR covid-19 OR disease-control OR public OR preventive OR preventative)):ti,ab,kw,de
	3		trust:ti
	4		‘public health’:ti
	5		#3 OR #4
	6		#1 AND #2 AND #5 AND [english]/lim AND ([article]/lim OR [article in press]/lim OR [data papers]/lim OR [editorial]/lim OR [erratum]/lim OR [letter]/lim OR [note]/lim OR [short survey]/lim)
